# Enhanced co-production of extracellular biopolymers and intracellular lipids by *Rhodotorula* using lignocellulose hydrolysate and fish oil by-product urea

**DOI:** 10.1186/s13068-025-02664-z

**Published:** 2025-06-11

**Authors:** Dana Byrtusová, Boris Zimmermann, Achim Kohler, Volha Shapaval

**Affiliations:** 1 Faculty of Science and Technology, Norwegian University of Life Sciences, Postbox 5003, 1432 Ås, Norway; 2 Drøbakveien 31, 1433 Ås, Norway

**Keywords:** *Rhodotorula*, Extracellular biopolymers, Renewable substrates, Lipids, Biomass, Lignocellulose

## Abstract

**Background:**

A key objective in microbial biorefinery technologies is to identify resilient microorganisms capable of simultaneously synthesizing diverse bioactive metabolites. Among these, *Rhodotorula* yeasts emerge as promising candidates for converting various waste streams and by-products into high-value chemicals. Their industrial potential stems from their ability to accumulate significant amounts of lipids and carotenoids while also secreting extracellular polymers such as exopolysaccharides, polyol esters of fatty acids, glycolipids, and enzymes—many of which remain to be fully characterized.

**Results:**

Among the five *Rhodotorula* strains tested, three exhibited substantial exopolysaccharide production. Notably, *Rhodotorula graminis* CCY 20-2-47 strain was identified, for the first time, to produce two distinct extracellular biopolymers—exopolysaccharides or polyol esters of fatty acids—depending on the growth conditions. It was observed enhanced production of exopolysaccharides up to 7.2 g L^−1^ and 14.7 g L^−1^ lipid-rich biomass by *Rhodotorula graminis* CCY 20-2-47 using lignocellulose hydrolysate and urea by-product. This study, for the first time, reports triggering effect of Mn^2+^ on exopolysaccharide production in *Rhodotorula*. Glucose-based medium resulted in co-production of polyol esters of fatty acids (3.9 g L^−1^) and lipid-rich biomass (15 g L^−1^) for *Rhodotorula graminis* CCY 20-2-47. Batch bioreactor fermentation for *Rhodotorula graminis* CCY 20-2-47 resulted in production of 13.1 g L^−1^ of exopolysaccharides and 50% w/w intracellular lipids when using lignocellulose hydrolysate and urea by-product. In contrast, 7.4 g L^−1^ of polyol esters of fatty acids and 35% w/w intracellular lipids were produced by the same strain on medium with pure glucose.

**Conclusions:**

In conclusion, *Rhodotorula* yeasts demonstrate significant potential for microbial biorefineries due to their ability to efficiently convert diverse waste substrates into valuable biomaterials, including lipids and extracellular biopolymers. This study provides new insights into a potential metabolic switch in extracellular polymer biosynthesis, driven by Mn^2+^ availability in the culture medium.

**Graphical abstract:**

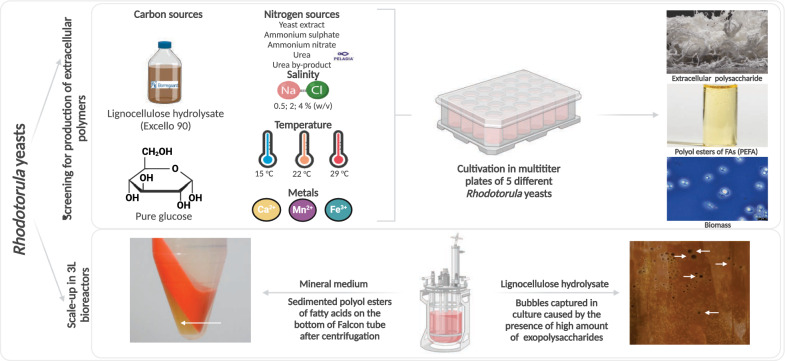

**Supplementary Information:**

The online version contains supplementary material available at 10.1186/s13068-025-02664-z.

## Highlights

- Lignocellulose hydrolysate induced biosynthesis of exopolysaccharides at *Rhodotorula* yeasts
- Manganese triggers production of exopolysaccharides at *Rhodotorula* yeasts
- Synthetic media without manganese resulted in biosynthesis of PEFA by one *Rhodotorula* strain
- Two extracellular metabolites can be produced by one *Rhodotorula* strain

**Supplementary Information:**

The online version contains supplementary material available at 10.1186/s13068-025-02664-z.

## Background

Carotenogenic or “red” yeasts represent biotechnologically promising microorganisms due to their ability to produce substantial quantities of intracellular lipids, predominantly acylglycerol oils and carotenoid pigments. Species from the genus *Rhodotorula*, including *Rhodotorula glutinis*, *R. mucilaginosa*, *R. graminis*, and *R. toruloides* are well-studied in this context. These yeasts are widely distributed in diverse environments such as soil, freshwater, saline water, plants, and glaciers [1, 2]. Characterized by high metabolic versatility, *Rhodotorula* species can utilize a wide array of substrates, including mono- and polysaccharides, glycerol, urea, inorganic and organic nitrogen sources, and fats [3]. This metabolic adaptability highlights their potential as efficient production platforms for flexible biorefineries capable of converting residual materials, by-products, and biowaste into a range of valuable bioproducts [1, 4–6].

Research on *Rhodotorula* predominantly focuses on lipid production, as these yeasts can accumulate up to 76% of their dry cell weight (% w/w) as lipids, primarily in the form of triacylglycerols (TAGs). The predominant fatty acids include palmitic (C16:0), stearic (C18:0), oleic (C18:1n9c), linoleic (C18:2n6c), and α-linolenic acid (C18:3n3) [7–12]. Beyond lipids, *Rhodotorula* produces also other valuable biomolecules such as carotenoids, natural pink-red pigments primarily composed of β-carotene, γ-carotene, torulene, and torularhodin [8]. These profiles vary significantly across species, strains, and growth conditions [13, 14]. Further, *Rhodotorula* biomass is rich in glucans [7]. Along with intracellular lipids and carotenoids, *Rhodotorula* have been reported to produce extracellular metabolites which could be polysaccharides, polyol lipids or enzymes [15–17].

The production of extracellular polysaccharides (EPS) has been extensively studied in bacteria, with notable examples including xanthan, gellan, dextran, and hyaluronan. In contrast, fungal EPS production has received less attention, although a wide variety of yeast genera have been reported to produce EPS, such as *Hansenula*, *Aureobasidium*, *Bullera*, *Debaryomyces*, *Lipomyces*, *Pichia*, *Candida*, *Trichosporon*, *Pseudozyma*, *Rhodotorula*, *Kluyveromyces*, *Hanseniaspora*, *Metschnikowia*, *Sporobolomyces*, *Phomopsis*, *Exophiala*, *Tremella*, *Kazachstania*, *Clavispora*, and *Cryptococcus* [18, 19]. Investigating EPS biosynthesis in *Rhodotorula* is particularly valuable not only because of the broad applications of EPS but also due to the high commercial potential of *Rhodotorula* yeast biomass itself. Previous studies have shown that EPS produced by *Rhodotorula* can be either homopolymers or heteropolymers composed of glucose, mannose, galactose, fucose, xylose, and/or uronic acids, with the composition and yield being strain-dependent [20–22]. The production of EPS depends on many factors, such as medium composition, temperature, pH and oxygen [20]. The reported EPS amounts can vary considerably, from below 1 g L^−1^ to over 28 g L^−1^ by *R. mucilaginosa*, *R. glutinis*, *R.* sp., and *R. toruloides* [20–22]. The application studies are focused on the use of EPS as healing agents in pharmacy, and as gelling and thickening agents in food-processing industries. Moreover, they have potential to be used as adjuvants to enhance nonspecific immunity, as hydrophilic matrix for development of vaccines and controlled release of drugs in pharmaceutical industry [20–22]. The separation of yeast-derived EPS is generally faster and less complex than in the case of bacterial and algal EPS, offering a major advantage for large-scale industrial production. Yeast-derived EPS are typically secreted directly into the surrounding medium during fermentation, facilitating straightforward recovery through processes like ethanol precipitation and centrifugation. In contrast, many cyanobacterial (algal) and bacterial EPS are tightly associated with the cell surface, often forming capsular structures. These capsular EPS require more intensive extraction methods, such as hot water treatments, chemical agents like ethylenediaminetetraacetic acid or sodium hydroxide, and physical disruption techniques, to release the polysaccharides [15, 21, 52, 53]. Moreover, the use of *Rhodotorula* offers a unique advantage by enabling simultaneous production of EPS, intracellular lipids, and carotenoids. This multi-product capability not only improves overall process efficiency, but also enhances the economic viability of such biotechnological production systems. By integrating the generation of high-value compounds within a single fermentation process, *Rhodotorula* presents significant potential for the development of sustainable and cost-effective biorefineries.

In addition, *Rhodotorula* species have also been shown to produce extracellular polyol esters of fatty acids (PEFAs), which consist of a polyol head group (commonly d-mannitol or d-arabitol) bound through the carboxyl end of an acetylated (*R*)-3-hydroxy fatty acid, typically saturated fatty acids such as C16:0 or C18:0 [16, 23–25]. Their production varies between below 1 g L^−1^ to over 48.5 g L^−1^, mainly by species of *R. paludigena*, *R. taiwanensis*, *R. kratochvilovae*, *R. babjevae*, and *R. glutinis* [5, 16, 23, 24, 26]. As substrates, lignocellulose hydrolysate, food hydrolysates, molasses, glycerol and inulin were reported [23, 27]. Due to their structural properties, PEFAs exhibit potential as biosurfactants, offering lower ecotoxicity, higher biodegradability, and improved activity across a broader range of environmental conditions compared to their synthetic counterparts [16, 23]. Some literature proposes using PEFA as an emulsifying or anticancer agent [2].

In the presented study, five carotenogenic EPS-producing yeasts from *Rhodotorula* genera were selected to test the induction of biosynthesis of extracellular polymers—EPS and PEFA. Initial screening in microtiter plates assessed the effects of media composition, temperature, salinity, and metal ions on the production of these metabolites. Together with synthetic media, two renewable by-products were selected in this study: lignocellulose hydrolysate and urea by-product from the fish oil industry. Additionally, the best-performing yeast and media, composed of by-products or pure components, were subsequently scaled-up in a 3-L bioreactor. The resulting EPS and PEFA were characterized by Raman and infrared spectroscopies.

## Methods

### Yeast strains and substrates

Five carotenogenic Basidiomycetes yeasts from genus *Rhodotorula*, obtained from the Culture Collection of Yeast (CCY, Institute of Chemistry, Slovak Academy of Science, Bratislava, Slovakia) and Culture Collection University of Gothenburg (CCUG, Gothenburg, Sweden) were used in the study. A detailed list of the yeast strains is presented in Table 1. The strains were stored at −80 °C in 1:1 mixture of 50% (*v*/*v*) glycerol and 24-h-old liquid culture grown in Yeast Extract-Peptone-Dextrose (YPD) broth (yeast extract, 10.0 g L^−1^; peptone, 20.0 g L^−1^; glucose 20.0 g L^−1^) (Merck, Darmstadt, Germany).

**Table 1:** List of the *Rhodotorula* yeasts used in the study

Code^a^	Yeast strain	Strain collection number
RG1	*Rhodotorula graminis*	CCY 20-2-47
RG2	*Rhodotorula graminis*	CCY 20-2-45
RG3	*Rhodotorula glutinis*	CCY 19-4-10
RG4	*Rhodotorula glutinis*	CCUG 32821
RG5	*Rhodotorula glutinis*	CCUG 48271

^a^Code used to report the results for different yeasts

Two substrates were used in the study: (1) commercial glucose-rich lignocellulose hydrolysate Excello-90 (Borregaard ASA, Norway) and (2) urea by-product from fish oil production (Pelagia AS, Norway). The Excello-90 lignocellulose hydrolysate was provided by Borregaard ASA (Sarpsborg, Norway). Borregaard employs a patented enzymatic pretreatment process specifically developed for Norwegian spruce biomass [54]. The resulting solid residue, mainly composed of cellulose, is subsequently hydrolysed into fermentable sugars using blends of cellulolytic enzymes. The resulting lignocellulose hydrolysate (Excello-90) is of high quality and contains low to no amount of inhibitors. All substrates were kept at 4 °C until use. The detailed chemical composition of the substrates is presented in Additional file 1, in Additional files section.

### Media and growth conditions

#### Screening cultivation in Duetz microtiter plates

Yeast strains were recovered from cryopreserved stock cultures by inoculation on YPD agar (yeast extract, 10.0 g L^−1^; peptone, 20.0 g L^−1^; glucose 20.0 g L^−1^; agar, 20.0 g L^−1^) (Merck, Darmstadt, Germany) and cultivated for 72 h at 22 °C. Inoculum was prepared by transferring one 10-μL loop of yeast biomass from YPD agar into 50 mL of sterile YPD broth. The cultivation was done in Erlenmeyer flasks for 24 h at 22 °C under constant shaking regime (130 rpm, 1.9 cm circular orbit) in the MAXQ 4000 incubator (Thermo Fisher Scientific, Waltham, MA, USA). The inoculum cultures were centrifuged (2330×*g*, 5 min and 15 °C) to remove the residual medium and re-suspended to the original volume with the sterile production medium. The inoculum was added to the sterile production medium in the ratio of 1:5 (*v*/*v*) and the cultivation was performed in Duetz Microtiter Plate System (Duetz-MTPS) [28–30] which consists of 24-well extra deep microtiter plates (MTPs) and sandwich covers mounted in the clamp systems onto the incubator shaking platform. The growth in production media took 96 h at 22 °C (if it is not stated otherwise) under the constant shaking regime (400 rpm and 1.9 cm circular orbit).

The following production media were used in the study: (1) high glucose-based media with different nitrogen sources: yeast extract (GYE), ammonium sulfate (GSO4), ammonium nitrate (GNO3), commercial urea (GCU), and urea by-product from fish oil production (GBU); (2) media based on lignocellulose hydrolysate Excello-90 as a carbon source and different nitrogen sources: yeast extract (EYE), ammonium sulfate (ESO4), ammonium nitrate (ENO3), commercial urea (ECU), and urea by-product from fish oil production (EBU) (Table 2). Based on the obtained results media GCU and EBU were selected to test the influence of sodium chloride (0.5, 2.0, and 4.0% w/v), temperature (15 and 29 °C), and metal ions (Ca^2+^, Mg^2+^ and Fe^3+^) on the production of exopolysaccharides and extracellular lipids. The detailed chemical composition of the production media is provided in Table 2. Metals added to the Excello-based media were selected according to the composition of the lignocellulose hydrolysate presented in Additional file 1, Additional files section.

**Table 2:** The detailed chemical composition of the production media used in the study

Chemical component (g L^−1^)	GYE	GSO4	GNO3	GCU	GBU	EYE	ESO4	ENO3	ECU	EBU
Glucose	52	52	52	52	52	–	–	–	–	–
Excello-90^a^	–	–	–	–	–	52	52	52	52	52
Yeast extract	2	–	–	–	–	2	–	–	–	–
(NH_4_)_2_SO_4_	–	0.99	–	–	–	–	0.99	–	–	–
NH_4_NO_3_	–	–	0.60	–	–	–	–	0.60	–	–
Urea	–	–	–	0.45	–	–	–	–	0.45	–
Urea by-product	–	–	–	–	0.72	–	–	–	–	0.72
KH_2_PO_4_	4	4	4	4	4	4	4	4	4	4
MgSO_4_·7H_2_O	0.7	0.7	0.7	0.7	0.7	0.7	0.7	0.7	0.7	0.7

Metals addition^b^	GCU or EBU medium						
CaCl_2_·2H_2_O	0.93 g L^−1^	–	–				
MnSO_4_·5H_2_O	–	7.76 mg L^−1^	–				
FeCl_3_·6H_2_O	–	–	6.05 mg L^−1^				

^a^Glucose equivalent—amount of Excello-90 diluted to reach the glucose concentration of 52 g L^−1^ (about 105 mL/L of Excello-90)

^b^Metals were added to either GCU or EBU media separately to test their individual effects on EPS and ELP production

#### Bioreactor scale-up

The bioreactor scale-up was done in a batch mode in two replicates using 3-L glass bioreactors (Minifors 2, Infors HT, Bottmingen, Switzerland) equipped with two six-bladed Rushton impellers and with maximum working volume of 2 L. The lignocellulose hydrolysate Excello-90 or pure glucose (final glucose concentration of 52 g L^−1^) were autoclaved in bioreactor at 121 °C for 15 min. Sterile nitrogen source (urea by-product, 0.72 g L^−1^) and salts (KH_2_PO_4_, 4 g L^−1^ and MgSO_4_⋅7H_2_O, 0.7 g L^−1^) were added aseptically by sterile syringe. The bioreactors were inoculated with 10% (*v*/*v*) 24-h-old yeast pre-culture and the cultivation was performed at 22 °C for 120 h. pH was set to 6.5 and maintained by pH probe (Hamilton, Switzerland) and automatic addition of 5 M KOH and 5 M H_2_SO_4_. Dissolved oxygen (DO) was kept above 30% of medium saturation (monitored by pO_2_-probe Hamilton, Switzerland) and the agitation rate was variable between 400 and 700 rpm. The foam was controlled by addition of Clerol antifoam (Carvin, France). Data were recorded by the eve bioprocess platform software (Infors HT, Bottmingen, Switzerland). During the fermentation, samples were collected regularly for the estimation of biomass concentration, extracellular lipids and EPS quantification and analysis of glucose consumption.

### Biomass preparation for further analysis

After cultivation in Duetz-MTPS and bioreactors, yeast biomass was centrifuged at 2330×*g*, 5 min, 15 °C for 5 min at 15 °C, and the biomass pellet was then washed three times using 0.1% NaCl solution. Further, yeast biomass was freeze-dried for 72 h and, subsequently, stored at −20 °C until use.

### Extraction and quantification of exopolysaccharides and polyol esters of fatty acids

Procedure for EPS isolation was adapted from Li et al. [22], with minor modifications. For each sample, the EPS containing supernatant collected after centrifugation of cell culture was treated with isopropanol in 2:1 volume ratio (isopropanol:supernatant). The suspension containing precipitated EPS was vortexed and centrifuged (1000×*g*, 3 min and 4 °C), and the residual supernatant was discarded. The EPS was washed two times with pure isopropanol. Finally, the resulting crude EPS was freeze-dried, and weight was measured using analytical balance.

PEFA fraction was extracted according to Guefali et al. [16] with some modifications. 4 mL of ethyl acetate were added to the washed biomass. The samples were vortexed (20 s) and centrifuged (2330×*g*, 10 min, 4 °C). Ethyl acetate phase was transferred into clean glass tube and evaporated under nitrogen flow at 30 °C. The resulting PEFA fraction was freeze-dried, and weight was measured using analytical balance. The purified EPS and PEFA fractions were stored at −20 °C until further analysis.

### Fourier transform (FT) infrared and FT-Raman spectroscopies

Fourier transform infrared (FTIR) reflectance spectra of the EPS and PEFA were measured using a single reflectance-attenuated total-reflectance (SR-ATR) High-Temperature Golden Gate ATR Mk II accessory (Specac, Orpington, United Kingdom) coupled to a Vertex 70 FTIR spectrometer (Bruker Optik GmbH, Ettlingen, Germany). The FTIR-ATR spectra were recorded with a total of 32 scans, spectral resolution of 4 cm^−1^, and digital spacing of 1.928 cm^−1^, over the range of 4000–600 cm^−1^, using Blackman–Harris 3-Term apodization, and the horizontal SR-ATR diamond prism with a 45° angle of incident. Approximately 1 mg of EPS or extracellular lipid was placed onto the ATR crystal for each measurement in 3 replicates. A background (reference) spectrum was recorded using the sample-free setup before the start of each sample measurement. The OPUS software (Bruker Optik GmbH, Ettlingen, Germany) was used for data acquisition, instrument control, spectral conversion and correction.

FT-Raman spectra were measured in backscattering geometry using a Multi-RAM FT-Raman spectrometer (Bruker Optik GmbH, Ettlingen, Germany) equipped with a neodymium-doped yttrium aluminum garnet (Nd:YAG) laser (1064 nm, 9394 cm^−1^), germanium detector cooled with liquid nitrogen, and a high-throughput screening (HTS) stage. Approximately 10 mg of sample was transferred into a 0.4 mL flat bottom glass insert vial and placed into a 96-well microplate. The plate was then placed in a HTS stage, and the laser was focused on the bottom of the vial. The FT-Raman spectra were recorded with a total of 512 scans, using Norton–Beer medium apodization, spectral resolution of 4 cm^−1^, and digital spacing of 1.928 cm^−1^, over the range of 3785–48 cm^−1^, at 1000 mW laser power. Each sample was measured in three technical replicates. The OPUS software (Bruker Optik GmbH, Ettlingen, Germany) was used for data acquisition, instrument control, and baseline correction.

### Total lipid content and fatty acid methyl esters profile

Lipid extraction was performed by a modified Lewis-method [31]. 15 ± 3 mg of freeze-dried yeast biomass was added into 2 mL polypropylene (PP) tubes together with 250 ± 20 mg acid-washed glass beads (710–1180 μm diameter, Sigma-Aldrich, USA) and 500 μL methanol. For disruption of yeast cells, the Precellys evolution homogenizer (Bertin Instruments, Germany) was used with shaking cycles of 5500 rpm (3 × 20 s). The content of the PP tube was transferred into a glass reaction tube by washing it with a 2.4 mL solvent mixture of methanol:chloroform:hydrochloric acid (7.6:1:1 *v*/*v*). 1 mg of tridecanoid acid (C13:0) in the form of TAG was used as an internal standard and added to the mixture. The glass tube was vortexed for 10 s and incubated for 1.5 h at 90 °C. After cooling to room temperature, 1 mL of distilled water and 2 mL hexane:chloroform (4:1 *v*/*v*) mixture was added. The separated upper hexane phase with extracted lipids was evaporated under nitrogen at 30 °C followed by the addition of sodium sulfate and dissolving the fatty acid methyl esters (FAMEs) in 1.5 mL hexane containing 0.01% butylated hydroxytoluene (BHT, Sigma-Aldrich, USA). Hexane containing extracted lipids were transferred into glass vials for GC analysis. Total lipid content (wt% of total FAMEs of the dry weight) and the fatty acid profile were estimated by 7820A gas chromatograph, Agilent Technologies equipped with an Agilent J&W GC column (20.0 m × 180 μm × 0.20 μm) and flame ionization detector (FID). 1 μL of the sample was injected in split mode (30:1 split ratio) to an inlet tempered to 280 °C. The total time of the analysis was 36 min with an initial temperature of 70 °C, which was kept for 2 min, and then increased by 10 °C/min to 150 °C, and finally by 6 °C/min to 230 °C. FAME standard mixture (C4–C24; Sigma Aldrich, USA) dissolved in hexane was used for the identification of the FAMEs. Quantification was based on the C13:0 internal standard.

### Analysis of residual glucose in media by GOPOD reagent

d-glucose Assay Kit—GOPOD Format (K-GLUC, Megazyme, Ireland) was used to determine glucose in lignocellulose hydrolysate and residual glucose in media in the bioreactor scale-up experiments. All reagent solutions and suspensions were prepared according to the manual provided by the producer (Megazyme, Ireland). 3 mL of GOPOD reagent was added to 0.1 mL of sample solution containing d-glucose and incubated at 40 °C for 20 min. After cooling to laboratory temperature, absorbance at 510 nm was recorded against the reagent blank composed of 3 mL of GOPOD and 0.1 mL of water. Glucose concentration was recalculated according to the calibration curve of d-glucose standard, recorded in the same way as the samples.

## Results

### Biomass, EPS, and PEFA concurrent production using different substrates

In total, ten production media with high C:N ratio were used to screen the selected set of EPS-producing *Rhodotorula* yeast. The chemical composition of the production media was based on the previously reported optimization experiments for achieving lipid-rich biomass [5, 7].

**Fig. 1:**
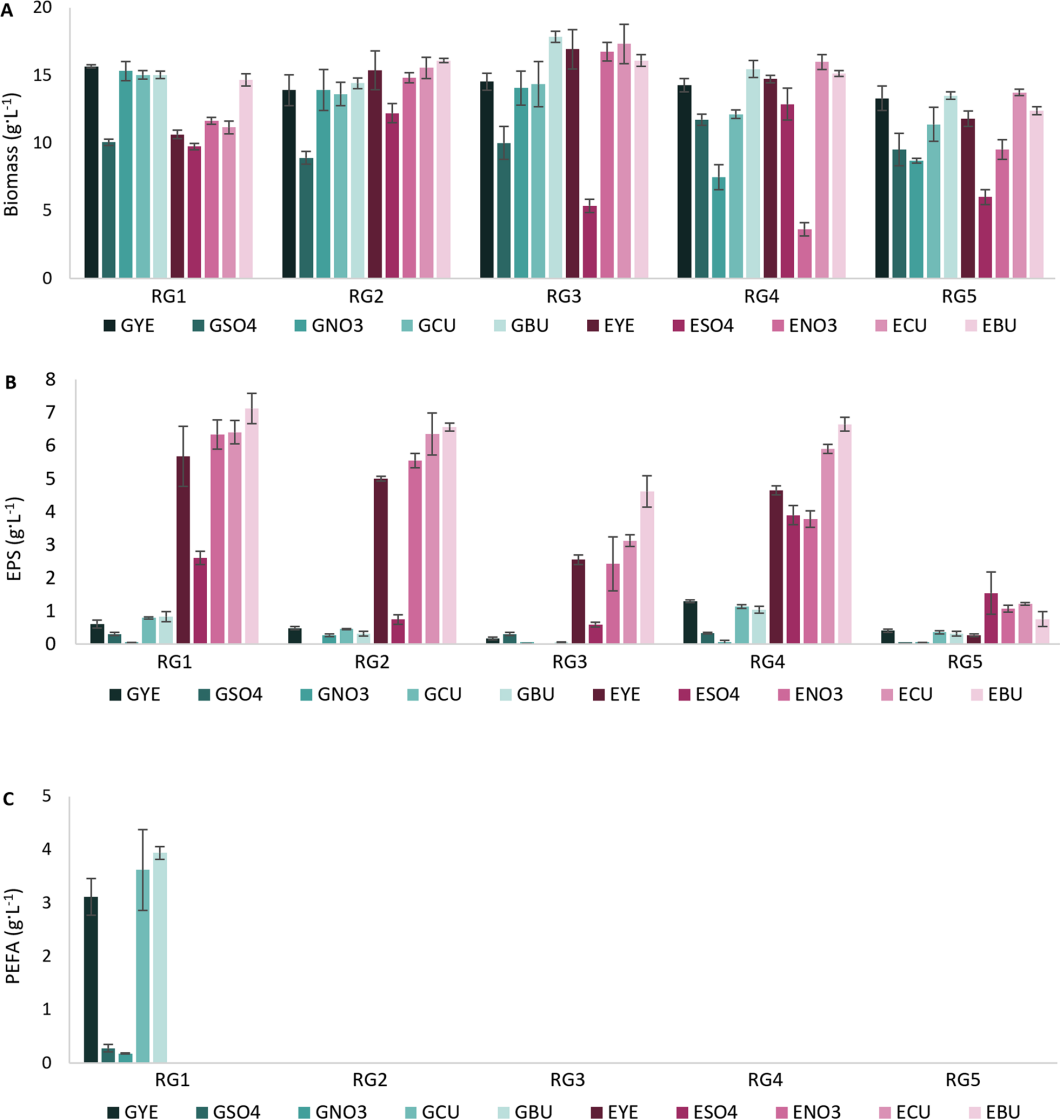
Biomass (**A**), exopolysaccharides (EPS) (**B**), and polyesters of fatty acids (PEFA) (**C**) production using glucose (GYE, GSO4, GNO3, GCU, and GBU) and lignocellulose (EYE, ESO4, ENO3, ECU, and EBU) media with different nitrogen sources. The absence of columns in the figure corresponds to the undetected production of EPS or PEFA

The biomass production using glucose-based media varied from 7.5 to 17.8 g L^−1^ and from 3.6 to 17.3 g L^−1^ when using lignocellulose-based media (Fig. 1A). Overall, commercial yeast extract, urea and urea by-product combined with glucose or lignocellulose hydrolysate Excello-90 (GYE, EYE, GCU, ECU, GBU, EBU) provided the highest biomass productivity for all *Rhodotorula* yeasts. RG1 showed lower biomass production on the media with lignocellulose, except for the medium where lignocellulose was combined with urea by-product (EBU) (Fig. 1A). Glucose-based media containing ammonium sulfate (GSO4) as nitrogen source provided the lowest biomass productivity for RG1-3 and RG5, while ammonium nitrate (GNO_3_) resulted in a similar observation for RG4-5. Similar results were obtained for lignocellulose-based media with these nitrogen sources (Fig. 1A).

In term of EPS production, remarkable differences can be seen between cultivation on glucose- and lignocellulose-based media (Fig. 1B). Overall, cultivation on lignocellulose hydrolysate media resulted in a higher EPS production than on glucose-based media, with total EPS content reaching over 6 g L^−1^ for RG1, RG2 and RG4 (Fig. 1B). The maximum EPS content on glucose-based media was 1.3 g L^−1^ for RG4 (Fig. 1C). To the author’s knowledge, this study is the first reporting of elevated EPS production in *Rhodotorula* when using lignocellulose hydrolysate. Cultivation on nitrogen sources, such as commercial yeast extract, urea and urea by-product, showed the highest EPS production for all yeast strains except RG3 and RG5. For these strains, glucose- and lignocellulose-based media with ammonium sulfate (GSO4), respectively, provided the highest EPS content (Fig. 1B). Similarly to biomass production, most of the studied *Rhodotorula* strains showed the lowest EPS production on the media with ammonium sulfate (GSO4 and ESO4) and, in some cases, with ammonium nitrate (GNO_3_ and ENO_3_) (Fig. 1B).

In the present study, RG1 was the only strain able to secrete PEFA when cultivated on glucose media (GYE, GCU and GBU) (Fig. 1C). Glucose combined with urea by-product (GBU) provided the highest amount of PEFA, 3.9 g L^−1^, followed by commercial urea and yeast extract, 3.6 and 3.1 g L^−1^, respectively. Conversely, the biosynthesis of PEFA was strongly inhibited when ammonium sulfate and ammonium nitrate were used. PEFA were not present in the media with lignocellulose hydrolysate.

### Impact of temperature, salinity, and metals on EPS and PEFA production

**Fig. 2:**
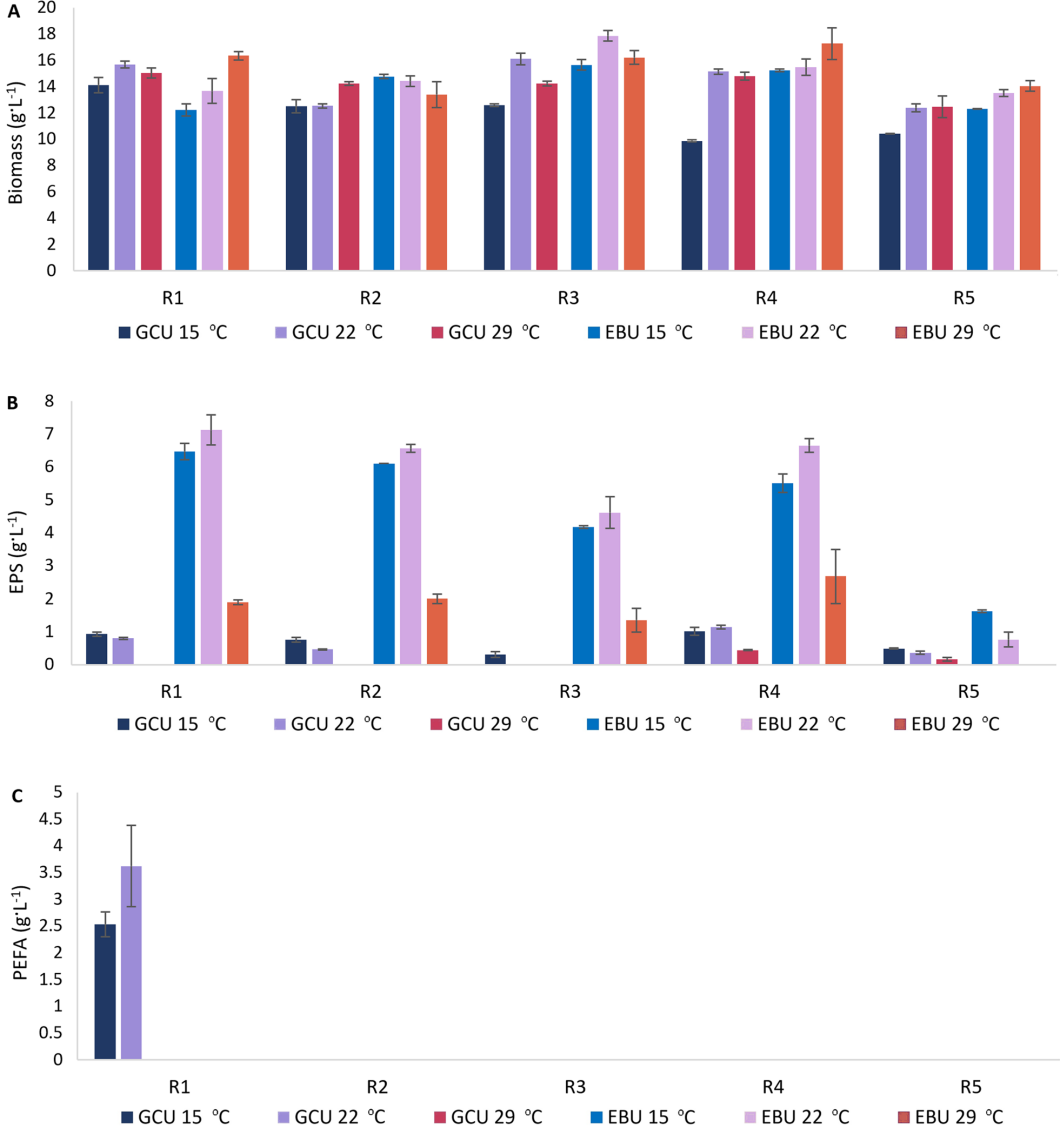
Biomass (**A**), exopolysaccharides (EPS) (**B**), and polyesters of fatty acids (PEFA) (**C**) production for *Rhodotorula* yeasts grown in GCU and EBU media at different temperatures. Absence of columns on the figure corresponds with not detected production of EPS or PEFA

**Fig. 3:**
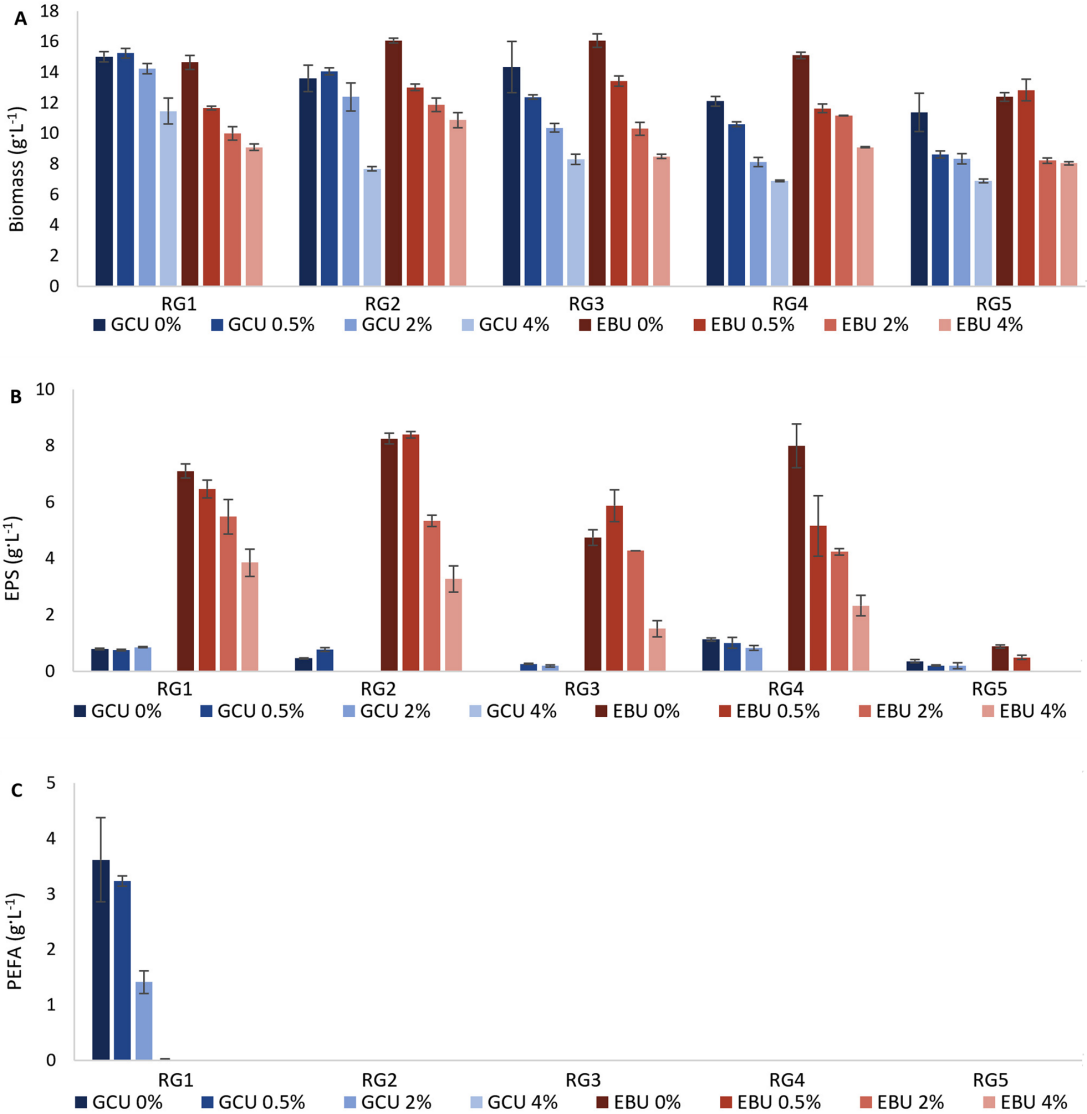
Biomass (**A**), EPS (**B**), and PEFA (**C**) production for *Rhodotorula* yeasts grown in GCU and EBU with different NaCl concentrations (0%, 0.5%, 2.0% and 4.0% (w/w)). Absence of columns on the figure corresponds with not detected production of EPS or PEFA

**Fig. 4:**
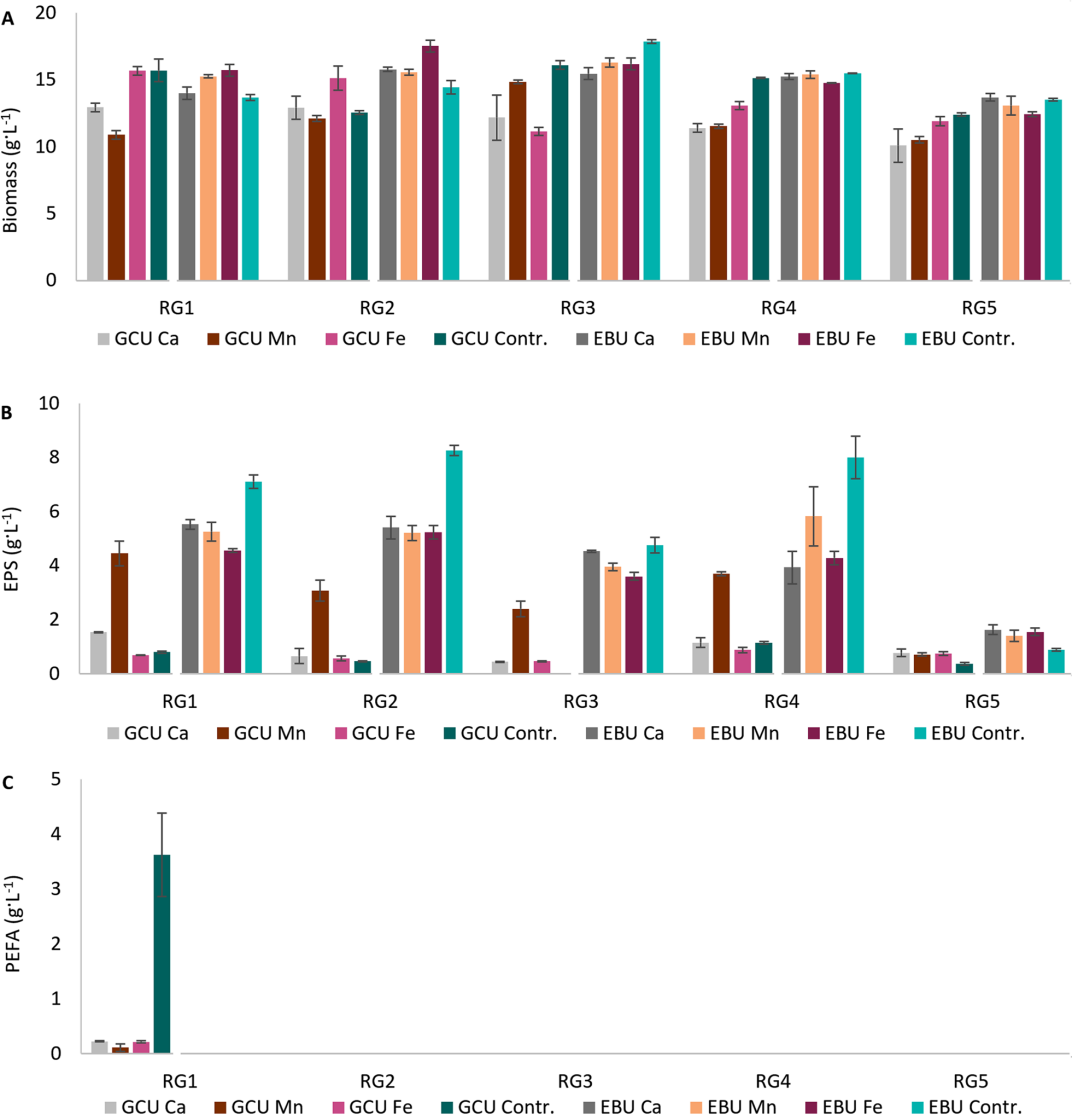
Biomass (**A**), EPS (**B**), and PEFA (**C**) production for *Rhodotorula* yeasts grown in GCU and EBU with different metals—Ca^2+^; Mn^2+^; Fe^3+^. Absence of columns in figure corresponds with not detected production of ESP or ELP

Since lignocellulose hydrolysate Excello-90 combined with urea by-product (EBU) was the best for producing biomass and extracellular biopolymers (EPS and PEFA) in *Rhodotorula*, it was selected for further optimization of EPS and PEFA production. In addition, glucose combined with commercial urea (GCU) was used as a control and PEFA production medium. The impact of temperature (Fig. 2), salinity (Fig. 3) and metal ions (Fig. 4) on EPS and PEFA production was investigated for all selected *Rhodotorula* strains.

Two temperatures—15 °C and 29 °C, along with the standard growth temperature (22 °C), were used in this study (Fig. 2A). For the majority of the studied *Rhodotorula* yeasts, cultivation at lower temperature (15 °C) resulted in decreased biomass production, except for RG2 and RG4 grown on lignocellulose, where biomass production was similar to the control of 22 °C. The cultivation at 29 °C showed diverse effect on *Rhodotorula*, where increase in biomass production was observed for RG2 grown on GCU, and RG2, RG4, and RG5 grown on EBU. While RG1 and RG3 grown on GCU, and RG2 and RG3 grown on EBU showed slight decrease in biomass production at 29 °C, the biomass production for RG4 and RG5 grown on GCU was similar to the cultivation at 22 °C. Interestingly, strains RG3 and RG5 had similar responses to temperature when grown on GCU and EBU, while other strains responded differently to temperature when grown on glucose and lignocellulose.

Use of 29 °C for cultivation resulted in a decrease of EPS production in *Rhodotorula* yeasts (Fig. 2B). Increase of EPS production was recorded for RG1, RG2, and RG3 grown on GCU at 15 °C, and RG5 grown on both media, while decrease of EPS production was for RG4 on GCU, and RG1, RG2, RG3, and RG4 on EBU at 15 °C (Fig. 2B). In this study the most optimal temperature for simultanous high biomass and EPS production was 22 °C.

PEFA production was observed only for RG1 grown on GCU at 15 °C and 22 °C, where lower temperature caused decrease in PEFA biosynthesis (Fig. 2C). Cultivation of RG1 at 29 °C resulted in a complete inhibition of PEFA production.

Supplementing the media with sodium chloride (NaCl) at various concentrations (0.5, 2.0 and 4.0% (w/v)) was tested to investigate the impact of salinity on the production of biomass and extracellular metabolites (Fig. 3). When NaCl was added to the glucose-based medium, it negatively impacted the biomass production for all yeast strains, where the biomass concentration was gradually decreasing along with the increase of NaCl concentration (Fig. 3A). Strain RG5 was able to tolerate 0.5% NaCl when grown on glucose and it responded with the considerable biomass decrease at all other concentrations. Similar results were recorded for RG3, RG4 and RG5 grown on lignocellulose media, while RG1 and RG2 were able to tolerate salt up to 2% of NaCl (Fig. 3A).

The production of EPS was either completely inhibited or significantly reduced in the presence of 4% NaCl. Interestingly, this inhibitory effect was observed when the strains were cultivated in a glucose-based medium. However, in a lignocellulose-based medium, EPS production still occurred, but at a reduced rate. This suggests that there are possibly other chemical components present in lignocellulose involved in mitigating the impact of salinity. The inclusion of 2% NaCl reduced EPS production in *Rhodotorula* strains grown on lignocellulose; however, the reduction was less severe compared to the effect of 4% NaCl (Fig. 3B). For the RG5 strain, which generally exhibits low EPS production, the presence of both 2% and 4% NaCl led to a complete inhibition of EPS synthesis when grown in a lignocellulose-based medium. For *Rhodotorula* strains cultivated on glucose-based medium, the addition of 2% NaCl inhibited EPS production in RG2 and reduced it in RG4 and RG5, whereas it enhanced production in RG1. Adding 0.5% NaCl caused a slight reduction in EPS production for RG1, RG4, and RG5 but stimulated EPS synthesis in RG2 and RG3. Interestingly, NaCl at 0.5% and 2% concentrations demonstrated a stimulatory effect on EPS production in certain *Rhodotorula* strains, highlighting strain-specific responses to salinity.

The biosynthesis of PEFA was significantly reduced with increasing NaCl concentrations (Fig. 3C). At 2% NaCl, PEFA production dropped to 1.5 g L^−1^, and at 4% NaCl, production was even more suppressed. This decline correlates with reduced biomass production and the maintenance of EPS biosynthesis observed at 0–2% NaCl.

Based on the chemical analysis of Excello-90 (Additional file 1, Additional files section), the following metal ions were identified as possible triggering agents for EPS production: calcium (Ca^2+^), iron (Fe^3+^), and manganese (Mn^2+^). The selected metal ions were added to the glucose combined with urea by-product medium at the same concentration as in Excello-90. In addition, the metal-containing salts at the same amount as in Excello-90 were added to EBU medium resulted in double concentration of selected metals in the hydrolysate. The addition of metal ions to glucose-based medium showed varying effects on biomass productivity and EPS production depending on the specific strain and type of metal ion (Fig. 4). For biomass productivity (Fig. 4A), strains RG1, RG4, and RG5 exhibited a decrease when supplemented with Ca^2+^ or Mn^2+^, while Fe^3+^ caused no change or only a slight reduction. In contrast, RG2 maintained stable biomass productivity with Ca^2+^ and Mn^2+^ but showed enhanced biomass production with Fe^3+^. RG3 displayed a notable decrease in biomass with the addition of Ca^2+^ and Fe^3+^, but Mn^2+^ caused only minor changes. When using Excello-90 medium with double the concentration of metal ions, there were no major effects on biomass production across all strains.

In terms of EPS production, Mn^2+^ supplementation in glucose-based media significantly enhanced output in all strains except RG5, which produced minimal EPS under all conditions. For example, EPS production in RG1 increased to 4.5 g L^−1^ with Mn^3+^ and 1.6 g L^−1^ with Ca^2+^, compared to 0.84 g L^−1^ in the control, representing more than a fivefold and twofold increase, respectively. Other metal ions in the glucose medium did not significantly alter EPS production, except of Ca^2+^ induction at RG1 and RG5. However, increasing the concentration of Ca^2+^, Fe^3+^, and Mn^2+^ in Excello-90 negatively affected EPS production in most strains except RG5. Notably, EPS production in Excello-90 was generally higher than in glucose-based media enriched with manganese. The reduced EPS production in EBU media additionally supplemented with metals likely resulted from the high metal concentration exerting toxic effects on cells. These findings suggest that optimization of growth media with a more gradual range of metal ion concentrations (specifically Mn and Ca) would be valuable to explore in future studies.

Addition of metal ions to glucose medium had negative effect on PEFA biosynthesis. The production decreased from 3.5 g L^−1^ (control medium—GCU) to below 0.5 g L^−1^ for all studied metals. The same as for EPS, there is no evidence of impact of metals on PEFA production. For manganese, the explanation could be switching to production of EPS by inhibiting PEFA biosynthesis. Generally, Ca, Mn, and Fe suppress the biosynthesis of PEFA. Therefore, the induction of EPS production by manganese could explain the absence of PEFA and the high EPS content in the growth medium based on lignocellulose hydrolysate.

### Fermentation in 3-L bioreactor

Fermentation experiment was done in a 3-L bioreactor for the most promising *Rhodotorula* yeast RG1 using GBU and EBU media, which were the most effective for producing EPS and PEFA, respectively. RG1 showed the highest EPS production on EBU (7.1 g L^−1^), and it was the only yeast strain capable of producing PEFA (Fig. 1).

**Fig. 5:**
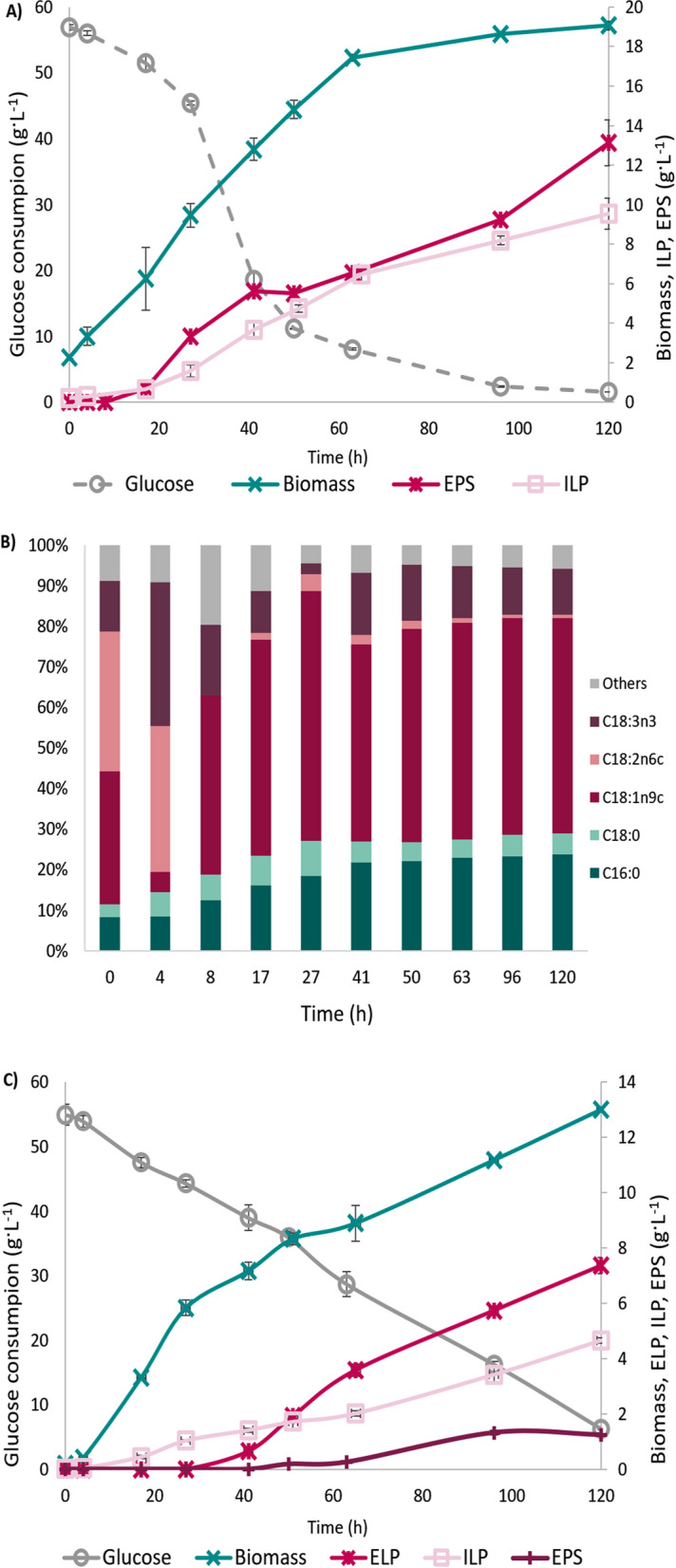

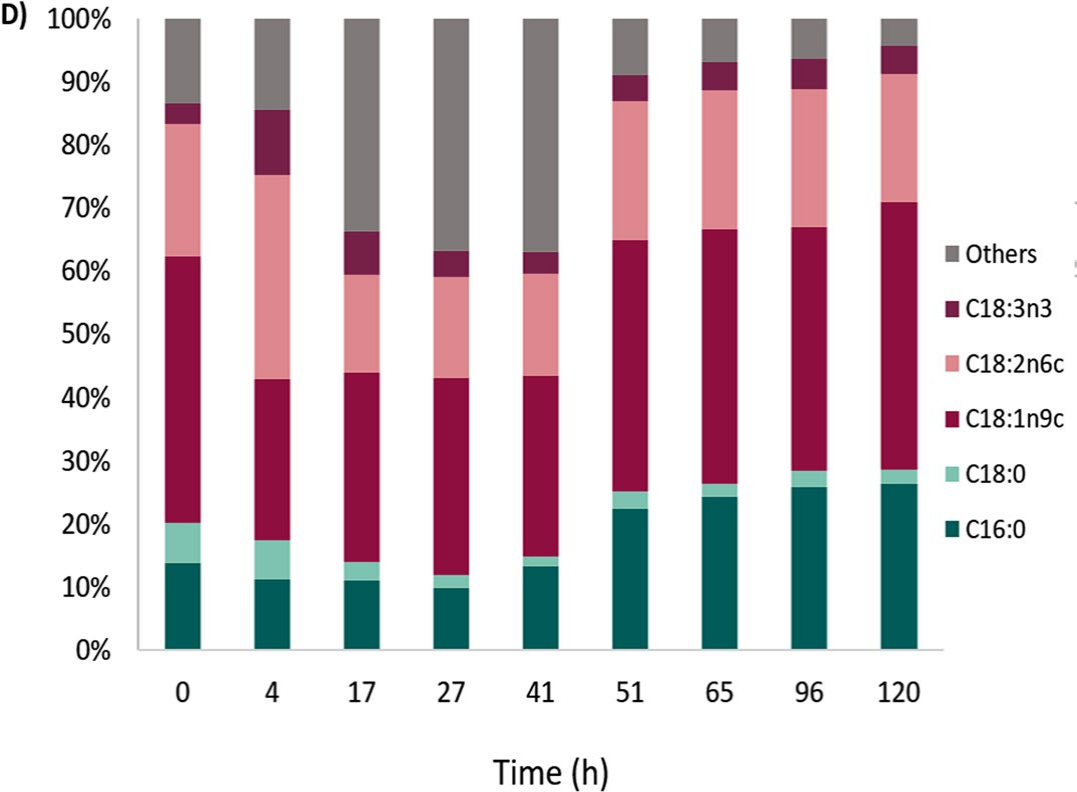
Biomass, EPS, intracellular lipids (ILP), and extracellular lipids (ELP—PEFA) production and glucose consumption at EBU medium (**A**), and GBU medium (**C**) in bioreactor. Fatty acids profile in EBU medium (**B**) and GBU medium (**D**)

As shown in Fig. 5A and Additional file 2, 19 g L^−1^ of yeast biomass containing 50% w/w of intracellular lipids (ILP), which corresponds to 9.5 g L^−1^, was produced along with 13 g L^−1^ of EPS when grown on the medium containing Excello-90 (EBU). An increase in biomass production was observed when lignocellulose hydrolysate Excello-90 was used as a carbon source. This can be attributed to the presence of other sugars in addition to glucose, such as xylose, mannose, and arabinose. In the literature [4] it has been reported that *Rhodotorula* can co-utilize these sugars alongside glucose, that may explain the increased biomass production observed when using Excello-90.

The simultaneous lipid accumulation and EPS biosynthesis started after 17 h of cultivation. After 48 h of cultivation the cell culture reached a high viscosity and air bubbles were observed due to the high production of EPS (Additional file 3). The culture reached stationary phase after 60 h, with a remaining glucose concentration of 10 g L^−1^. The biomass, EPS and ILP reached maximum at 120 h, by which time all the glucose had been consumed. According to GC-FID analysis, the main fatty acids of intracellular lipids are palmitic acid (C16:0), stearic acid (C18:0), oleic acid (C18:1n9c) and linoleic acid (C18:2n6c) (Fig. 5B).

For the medium containing only glucose, less substrate (commercial glucose) was consumed leading to lower biomass production (13 g L^−1^). The intracellular lipids content reached lower values compared to fermentation using lignocellulose hydrolysate (38% w/w). Simultaneous PEFA and EPS were detected after 27 h of cultivation, whereas intracellular lipids started to increase as early as 4 h in the bioreactor. The intracellular lipids are composed mainly of palmitic acid (C16:0), oleic acid (C18:1n9c), linoleic acid (C18:2n6c) (Fig. 5D). The glucose was not consumed after 120 h, leaving 6 g L^−1^ in the medium.

Overall, lignocellulose hydrolysate combined with urea by-product from fish industry have the potential to be used to produce EPS and yeast biomass with high lipid content. Manganese in Excello-90 induced EPS biosynthesis, which increased yield from 0.028 g_EPS_/g_S_ (GBU) to 0.235 g_EPS_/g_S_ and productivity from 0.010 g_EPS_/(L h) to 0.109 g_EPS_/(L h) (Table 3). Conversely, using substrate without manganese (pure glucose, GBU) reduces biosynthesis of EPS and induces secretion mainly of PEFA instead, with the yield of 0.166 g_ELP_/g_S_. This demonstrates the versatility and efficiency of the RG1 strain in utilizing different substrates for biosynthesis of a broad range of metabolites.

**Table 3:** Endpoint (120 h) biomass concentration, total fatty acids (TFA) content, EPS and ELP production and their productivity (Q) and yield (Y) from bioreactor cultivations of *R. graminis* CCY 20-2-47 (RG1) based on values from start and endpoint measurements

Parameters	Excello-90 (EBU)	Glucose (GBU)
Biomass (g L^−1^)	19.06 ± 0.22	13.01 ± 0.04
TFA (g L^−1^)	9.55 ± 0.79	4.66 ± 0.10
TFA (% w/w)	50.09 ± 3.55	35.80 ± 0.66
EPS (g L^−1^)	13.14 ± 1.17	1.25 ± 0.14
ELP (g L^−1^)	–	7.37 ± 0.29
Y_X/S_^a^ (g_X_/g_S_)	0.340 ± 0.011	0.273 ± 0.003
Y_ILP/S_^a^ (g_ILP_/g_S_)	0.167 ± 0.016	0.167 ± 0.001
Y_EPS/S_^a^ (g_EPS_/g_S_)	0.235 ± 0.026	0.028 ± 0.001
Y_PEFA/S_^a^ (g_PEFA_/g_S_)	–	0.166 ± 0.003
Q_X_^b^ (g_X_/(L h))	0.159 ± 0.002	0.108 ± 0.001
Q_TFA_^b^ (g_TFA_/(L h))	0.080 ± 0.007	0.039 ± 0.001
Q_EPS_^b^ (g_EPS_/(L h))	0.109 ± 0.010	0.010 ± 0.000
Q_PEFA_^b^ (g_PEFA_/(L h))	–	0.061 ± 0.002

^a^Yield of biomass (X)/ILP/EPS/ELP on consumed glucose (S), based on values from start and endpoint measurements

^b^Hourly biomass (X), ILP, EPS and ELP productivities, based on values from start and endpoint measurements

### Spectroscopy analysis of extracellular metabolites

**Fig. 6:**
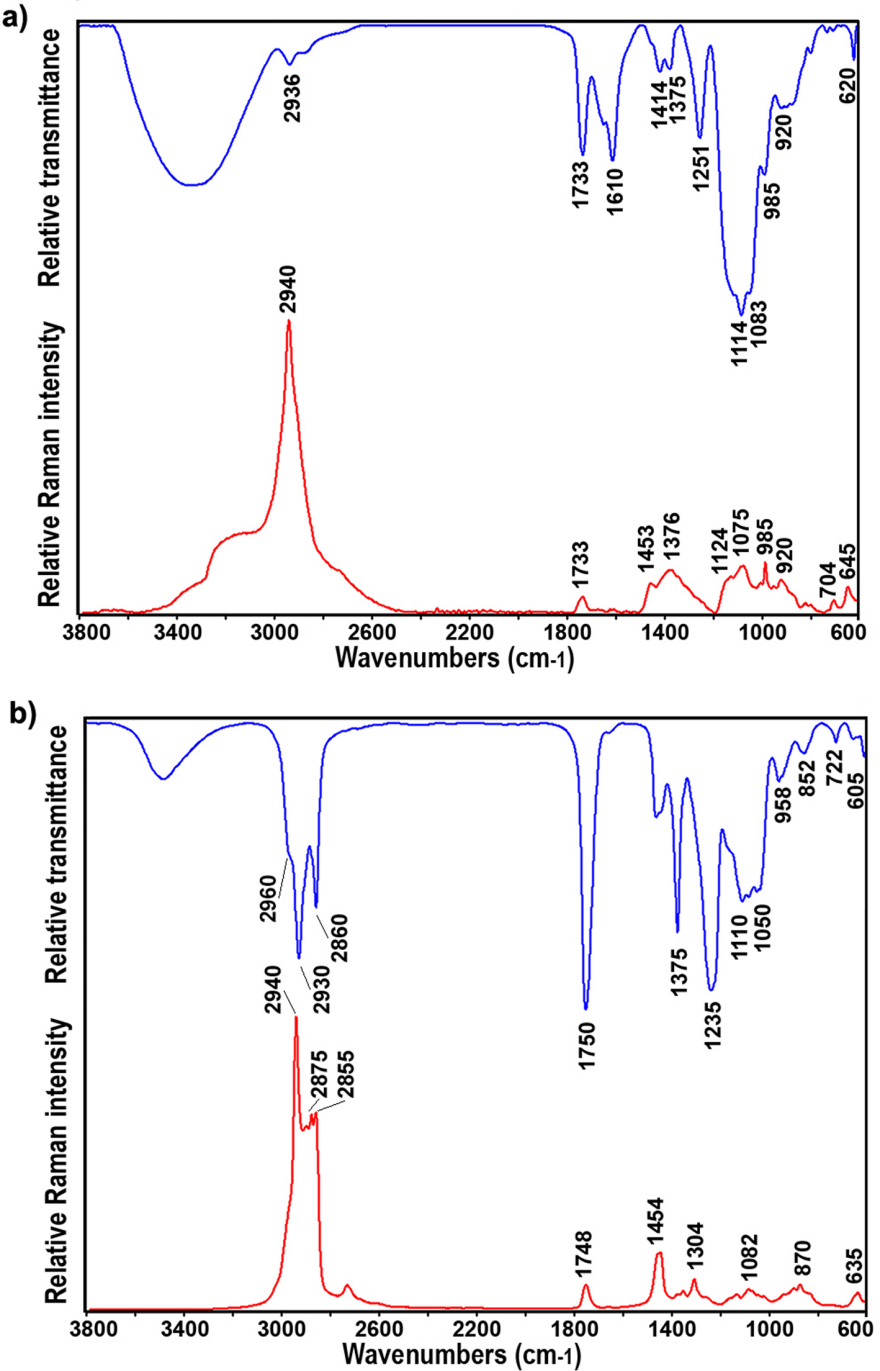
FT-IR (up, blue) and FT-Raman (down, red) spectra of: **a** exopolysaccharide (EPS), and **b** polyol ester of fatty acid (PEFA)

The present study focuses on the production of extracellular metabolites and does not deal with detailed chemical analysis of these products; a comprehensive chemical characterization study will be reported in the future. The preliminary chemical analysis was conducted using FT-IR and FT-Raman spectroscopies, as these analytical methods have proven indispensable for the rapid detection and characterization of *Rhodotorulla* metabolites [8]. These spectroscopies were used for the overall characterization and identification of functional groups in both extracellular metabolites: EPS obtained after cultivation on Excello-90 combined with urea by-product (EBU) and PEFA obtained from cultivation on glucose combined with urea by-product medium (GBU). The spectra are presented in Fig. 6. The presence of carbonyl group (C=O stretching at 1733 cm^−1^) in both FT-IR and FT-Raman spectra of EPS indicates that the EPS contains monosaccharides based on uronic acids (Fig. 6a). The vibrational spectra of EPS show peak at approx. 3300 cm^−1^ which corresponds to stretching vibration of hydroxyl (-OH) groups. The bands at 2940–2930 cm^−1^ in the spectra are related to C–H stretching vibrations of –CH_3_ and –CH_2_ groups, while the broad stretch region from 1000–1200 cm^−1^ corresponds to the C–O, C–C stretching, C–O–C and C–O–H deformation vibrations of polysaccharides [22, 32].

The spectra of PEFA (Fig. 6b) show C–H stretching vibrations of –CH_3_ and –CH_2_ groups at 2960–2850 cm^−1^, C=O stretching of ester groups (1750–1745 cm^−1^), CH_2_ and CH_3_ deformations (1460–1440, 1380–1350 cm^−1^), C–C stretching (1150–1050, in FT-Raman spectrum), C–O–C stretching (1235 and 1110–1050 cm^−1^, in FT-IR spectrum), and CH_2_ deformation (722 cm^−1^, in FT-IR spectrum) [16, 33]. The measured FT-IR spectra of PEFA correlate well with the results from Guerfali et al., where PEFA were identified as d-mannitol and d-arabitol esters of fatty acids with amphiphilic structure typical for glycolipids [16].

## Discussion

In total, ten production media with high C:N ratio were used to screen the selected set of *Rhodotorula* yeast for their ability to produce extracellular biopolymers (EPS and PEFA) along with the biomass. Based on the performance, EBU and GCU were selected to continue exploring the influence of temperature (15, 22, and 29 °C), salinity (0.5, 2.0 and 4.0% (w/v)), and impact of metal ions such as calcium (Ca^2+^), iron (Fe^3+^), and manganese (Mn^2+^). *Rhodotorula graminis* CCY 20-2-47 due to its best performance was selected and scaled-up in 3-L bioreactor.

Organic nitrogen sources, such as yeast extract, are widely recognized for their rich nutrient content [34, 35], a finding corroborated by our study. Ammonium sulfate, another commonly used nitrogen source [34, 35], showed to be unsuitable for lipid-rich biomass and extracellular metabolite co-production in our case. The low biomass yield observed with ammonium sulfate may be attributed to pH fluctuations in cultivation media. Upon uptake by yeast cells, NH_4_^+^ dissociates into NH₃, which is utilized for biomass formation. To maintain homeostasis, yeast cells actively export protons via plasma membrane H^+^-ATPases, leading to acidification of the medium [36]. In contrast, commercial urea supported biomass production as effectively as commercial yeast extract, while the urea by-product yielded the highest biomass accumulation. This may be linked to the presence of residual lipids, which could serve as an additional carbon source for certain *Rhodotorula* strains. Urea assimilation in yeast involves enzymatic hydrolysis by urease, generating NH_4_^+^ and CO_2_. The released ammonium is subsequently integrated into nitrogen metabolism via glutamine synthetase and glutamate synthase pathways [36].

To the best of the author’s knowledge, this study is the first to report enhanced EPS production in *Rhodotorula* utilizing lignocellulose hydrolysate. It has been reported previously that organic nitrogen sources, such as yeast extract, are one of the most efficient substrates to produce EPS by *Rhodotorula* yeasts [37]. Our study showed that for carotenogenic yeasts commercial urea, urea by-product and ammonium nitrate provide better EPS productivity than yeast extract, while according to the literature ammonium sulfate is the most often used [20]. Similar results were observed for glucose-based media, where ammonium sulfate provided higher EPS than ammonium nitrate, while opposite results were observed for lignocellulose-based media. Lignocellulose-based medium with urea by-product (EBU) showed to be the most optimal for EPS production for all yeast strains with exception of RG5, which had overall low EPS production on all tested media. Our study is the first to report induced EPS biosynthesis using lignocellulose hydrolysate combined with urea. To the author’s knowledge, there are no other studies reporting the impact of urea on the production of EPS in yeast or other microorganisms.

Different temperature responses of the studied *Rhodotorula* strains when grown on glucose and glucose-rich lignocellulosic hydrolysate can be attributed to both substrate-specific biochemical and physiological differences and intrinsic strain-specific metabolic adaptations. Glucose transporters, for instance, exhibit variations in affinity and thermal stability across yeast species and strains [38]. In lignocellulose hydrolysates, the presence of competing sugars and inhibitors can further affect glucose transport efficiency, with temperature playing a crucial role in modulating transporter function. Exello 90 lignocellulose hydrolysate is supplied by Borregaard, and contains only negligible levels of potential inhibitors. Cultivation on lignocellulosic hydrolysates often leads to the co-utilization of glucose and non-glucose sugars, with yeasts differing in the thermal sensitivity of key enzymes involved in non-glucose sugar metabolism (e.g., xylose reductase, xylitol dehydrogenase) [39]. However, such temperature-dependent enzymatic responses have not been extensively studied in *Rhodotorula*. Notably, a similar temperature effect was previously reported by Zhao and Li [40], where elevated temperatures negatively impacted EPS production. Several studies suggest that lower temperatures can stimulate EPS biosynthesis [20, 22, 40], an observation partially corroborated by our findings.

The molecular mechanisms underlying temperature-driven EPS production remain poorly understood. One possible explanation is that elevated temperatures may redirect metabolic fluxes away from secondary metabolite synthesis, including EPS. Additionally, high temperatures may impair key enzymatic activities essential for EPS biosynthesis [41] or disrupt membrane fluidity, thereby affecting sugar transport and precursor availability. Conversely, low temperatures might activate regulatory pathways that upregulate EPS biosynthesis genes as part of a cold stress response. Our findings suggest that PEFA biosynthesis in *Rhodotorula* is highly sensitive to temperature fluctuations. This sensitivity could stem from the thermal instability of enzymes involved in PEFA synthesis and transport. Moreover, high temperatures may exert an inhibitory effect by promoting enzyme denaturation and oxidative degradation of fatty acid precursors.

The influence of NaCl on *Rhodotorula* growth and biomass production varied among the studied *Rhodotorula* species. Kot et al. [42] reported increased biomass production in *R. glutinis* and *R. mucilaginosa* but a decline in *R. gracilis* at 5% NaCl. Conversely, Yen et al. [43] observed reduced biomass production in *R. mucilaginosa* at NaCl concentrations ranging from 0.1 to 40 g/L. The production of EPS by *Rhodotorula* yeasts in saline conditions remains poorly documented, and its regulation appears multifactorial, depending on species, growth conditions, and metabolic pathways. EPS secretion is often an adaptive response to osmotic stress, functioning as a protective mechanism by balancing osmotic pressure and retaining water to maintain cell integrity [44]. However, the effect of NaCl on EPS production in *Rhodotorula* is inconsistent. While moderate salt concentrations may stimulate EPS synthesis, higher levels tend to inhibit it. For instance, Li et al. found no significant changes in EPS production by marine *R. mucilaginosa* across increasing NaCl concentrations. In our study, elevated NaCl concentrations (2% and 4%) generally suppressed EPS production, except in the RG1 strain grown in GBU medium at 2% NaCl [22]. This suggests that, in these strains, EPS biosynthesis may not primarily serve as an osmoadaptive response to high salinity.

The presence or absence of metal ions in the growth medium can induce shifts in biochemical pathways, influencing biopolymer accumulation. Metal ions play strain-specific roles in microbial metabolism, often serving as cofactors in enzymatic reactions, cellular metabolism, and oxidative stress management [45]. Metals are essential for various biochemical processes, typically as enzymatic cofactors. In yeast cells, Mn^2+^ is transported to the Golgi lumen, where it serves as a cofactor for glycosyltransferases—enzymes responsible for transferring sugar nucleotides (the building blocks of EPS) to acceptor molecules, potentially forming polymer chains [46, 47]. Additionally, manganese metalloproteins are involved in a broad spectrum of functions, including oxidoreductases, DNA and RNA polymerases, peptidases, kinases, and decarboxylases. However, the precise mechanisms of manganese homeostasis in yeast cells remain incompletely understood [47]. Other metals also play significant roles in microbial metabolism. Iron functions as a redox cofactor in numerous cellular processes, while calcium is crucial for cell signalling, influencing fungal growth, development, virulence, and stress responses. Calcium homeostasis is tightly regulated to maintain optimal concentrations in the cytosol and organelles [48]. The direct role of manganese in *Rhodotorula* EPS biosynthesis remains unclear and has not been previously reported in the literature. Our study, for the first time, demonstrates that the addition of manganese to a mineral medium significantly induced EPS synthesis, highlighting its critical role in *Rhodotorula* polysaccharide secretion.

Fermentation experiment in bioreactor for the most promising *Rhodotorula* yeast strain RG1 using GBU and EBU media, which were the most effective for producing EPS and PEFA, respectively. RG1 showed the highest EPS production on EBU, and it was the only yeast strain capable of producing PEFA. The production of EPS depends on many factors, such as *Rhodotorula* strain, medium, temperature, pH and oxygen. In literature, the amount could vary from below 1 g L^−1^ to over 28.5 g L^−1^ [20–22]. Li et al. [22] reached over 15 g L^−1^ of EPS by using glucose and yeast extract. Silambarasan et al. [49] produced 7.5 g L^−1^ of EPS by *Rhodotorula* sp. combining sucrose and yeast extract. Our results are among the highest reported. For the medium containing only glucose, EPS biosynthesis was inhibited and PEFA secretion into culture medium induced instead. Yeasts are known to produce glycolipids extracellularly. Some *Candida* strains can reach up to 300 g/L of glycolipids at continuous feeding [50]. *Rhodotorula* yeasts are not explored in such detail in this regard. The highest reported production of PEFA by *Rhodotorula* yeast is 48.5 g L^−1^ by *Rhodotorula paludigena* P4R5 after 156 h of inulin utilization [23]. Cajka et al. [51] generated 8.6 g L^−1^ of PEFA by *Rhodotorula babjevae* grown on the medium with 100 g L^−1^ of glucose. Guerfali et al. [16] reached 3.1 g L^−1^ of PEFA also with *Rhodotorula babjevae* when using glucose at high C:N ratio. In this study, the PEFA production reached over 7 g L^−1^ in simple batch fermentation where the EPS biosynthesis was six times less than by utilizing lignocellulose hydrolysate.

## Conclusions

This study highlights the potential of *Rhodotorula* yeasts to synthesize extracellular polymers using sustainable feedstocks. Our findings demonstrate the feasibility of utilizing lignocellulose hydrolysate and a fish industry by-product, urea, thereby supporting a circular economy and minimizing environmental impact. Beyond their well-established roles as sources of lipids and carotenoids, *Rhodotorula* species also exhibit promise for the production of extracellular polymers, including EPS and PEFA. Notably, this study is the first to report the simultaneous production of two distinct extracellular metabolites alongside lipid-rich biomass in carotenogenic yeasts. We found that metals present in lignocellulose hydrolysate, particularly Mn^2+^, induced EPS synthesis, whereas their absence redirected extracellular metabolism towards PEFA secretion. These findings provide new insights into a potential metabolic switch that regulates EPS biosynthesis, modulated by the availability of metals in the culture medium. This study also shows that *Rhodotorula* yeasts emerge as promising candidates for the concurrent production of EPS and lipid-rich, pigmented biomass, offering innovative, cost-effective, and environmentally sustainable biotechnological solutions.

## Supplementary Information

**Additional file 1.**

**Additional file 2.**

**Additional file 3.**

## Data Availability

No datasets were generated or analysed during the current study. All data generated or analysed during this study are included in this published article (and its additional files).

## Abbreviations

CDW: Cell dry weight
EPS: Exopolysaccharides
FAMEs: Fatty acids methyl esters
HTS: High-throughput screening
ILP: Intracellular lipids
MTPs: Microtiter plate system
MUFA: Monounsaturated fatty acids
PEFA: Polyol esters of fatty acids
PUFA: Polyunsaturated fatty acids
SAT: Saturated fatty acids
