# Supplementary material for: Enhanced co-production of extracellular biopolymers and intracellular lipids by Rhodotorula using lignocellulose hydrolysate and fish oil by-product urea
Source: Biotechnol Biofuels Bioprod. 2025 Jun 11;18:61. doi: 10.1186/s13068-025-02664-z (PMC12153088; doi:10.1186/s13068-025-02664-z)
Supplement: Supplementary file 1 — Additional file 1. [file 13068_2025_2664_MOESM1_ESM.docx]

**Additional files**

**Additional file 1:** Chemical composition of the commercial lignocellulose hydrolysate Excello-90 provided by Borregaard ASA (Norway), and urea by-product provided by Pelagia AS (Norway).

| **Component** | **Excello-90** | **Urea by-product** |
| --- | --- | --- |
| Dry matter (DM) | 63 % | - |
| Ash | 3.6 % on DM | - |
| Glucose | 495.2 g·L^-1^ | - |
| Xylose | 51.7 g·L^-1^ | - |
| Arabinose | 4.9 g·L^-1^ | - |
| Galactose | 4.9 g·L^-1^ | - |
| Mannose | 44.40 g·L^-1^ | - |
| Cellobiose | 20.6 g·L^-1^ | - |
| Gentiobiose | 24.7 g·L^-1^ | - |
| Fructose | 14.0 g·L^-1^ | - |
| Glycerol | 12.3 g·L^-1^ | - |
| p-coumaric acid | 10.26 ug·g^-1^ | - |
| Ferulic acid | 35.22 ug·g^-1^ | - |
| 5-hydroxymethylfurfural | 23.30 ppm | - |
| Furfural | 24.70 ppm | - |
| Lactic acid | 1.3 % on DM | - |
| Acetic acid | 1.6 % on DM | - |
| Formic acid | 0.5 % on DM | - |
| Ca | 0.183 % (w/w) | - |
| Mn | 12.8 mg/kg | - |
| Fe | 9.04 mg/kg | - |
| Na | 0.261 % (w/w) | - |
| Nitrogen | 0.07 g/100 g | 29.1 % (w/w) |
| Moisture | - | 11.9 % |
| Fat | - | 19.8 % (w/w) |

**Additional file 2 (tiff format):** Intracellular lipid content (ILP) (% of DCW) in yeast biomass grow in bioreactor on EBU and GBU medium.

**Additional file 3 (tiff format):** Picture of bubbles captured in the culture in bioreactor in EBU medium (A) and PEFA sedimented on the bottom of Falcon tube after centrifugation (B).
