# Supplementary figures and images for: Enhanced co-production of extracellular biopolymers and intracellular lipids by Rhodotorula using lignocellulose hydrolysate and fish oil by-product urea
Source: Biotechnol Biofuels Bioprod. 2025 Jun 11;18:61. doi: 10.1186/s13068-025-02664-z (PMC12153088; doi:10.1186/s13068-025-02664-z)

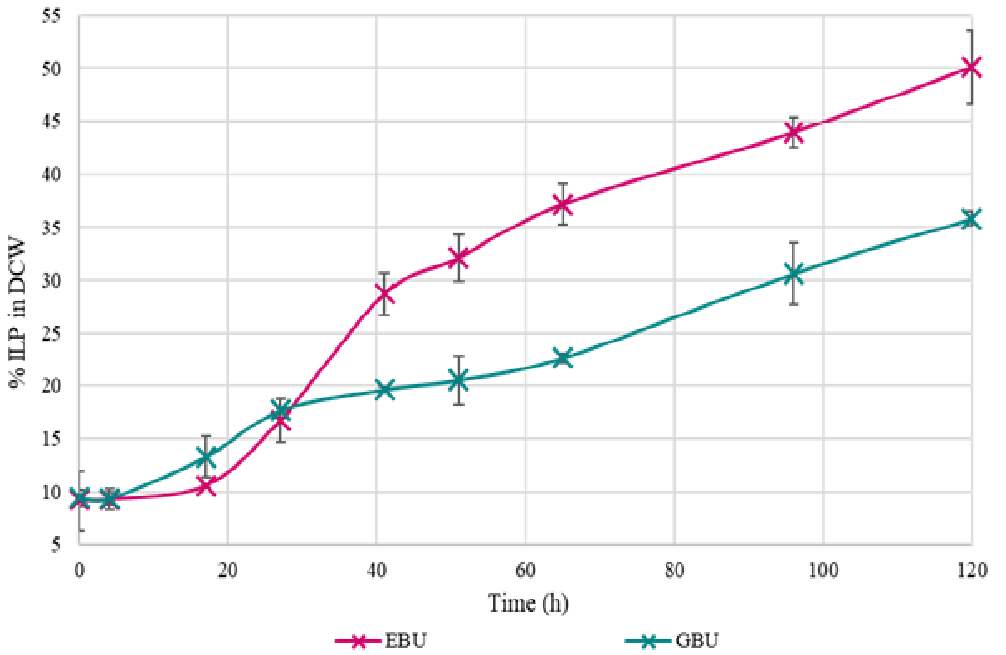

Supplement: Supplementary file 2 — Additional file 2. [file 13068_2025_2664_MOESM2_ESM.tif]

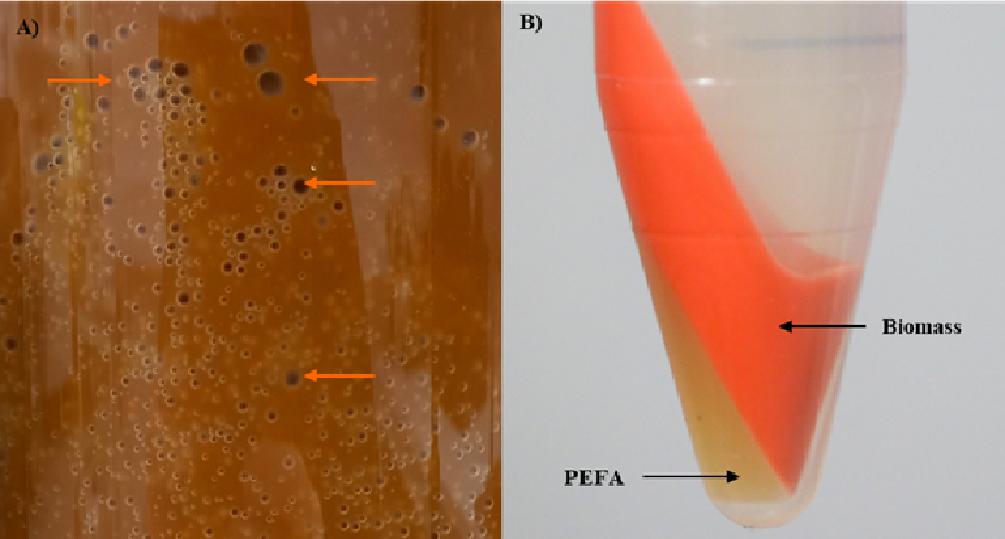

Supplement: Supplementary file 3 — Additional file 3. [file 13068_2025_2664_MOESM3_ESM.tif]
